# Experimental study and new three-dimensional kinetic modeling of foamy solution-gas drive processes

**DOI:** 10.1038/s41598-018-22669-z

**Published:** 2018-03-12

**Authors:** Xiaofei Sun, Yanyu Zhang, Shilin Wang, Zhaoyao Song, Peng Li, Changfa Wang

**Affiliations:** 1 China University of Petroleum (East China), School of Petroleum Engineering, Qingdao, 266580 PR China; 2 Shengli Oilfield, Luming Oil and Gas Exploration & Development Co. LTD., 257000, Dongying, PR China

## Abstract

Foamy solution-gas drive processes in heavy oil reservoirs are very complex. The influence of some microscopic factors on this process is not fully understood due to limitations of traditional depletion tests. This study aims to investigate foamy solution-gas drive by experiments and simulations. First, the effects of the pressure depletion rate on critical gas saturation and foamy solution-gas drive processes were investigated by laboratory experiments. Second, a new three-dimensional foamy oil model that captures many important characteristics of foamy solution-gas drive, such as non-equilibrium behavior, gas evolution kinetics, and the effect of viscous forces on gas mobility, was developed. Last, the effects of some important parameters on foamy solution-gas drive were systematically investigated,and a model application was conducted in a typical foamy oil reservoir. The results indicate that the new model is capble of simulating many of the unusual behaviors observed in foamy solution-gas drive on a laboratory and field scales. High oil recoveries were obtained with a high oil viscosity, high depletion rate, long sandpack, and low solution gas-oil ratio. Foamy solution-gas drive processes are sensitive to the depletion rate, length, and critical gas saturation. The oil viscosity, solution GOR and diffusion coefficient are not sensitive factors.

## Introduction

In solution gas drive reservoirs, gas is released from solution when the reservoir pressure is below the bubble-point pressure. Gas initially exists in the form of tiny bubbles which is immobile due to the existence of capillary forces. Gas flows only after the bubbles become sufficiently large to coalesce into a continuous gas phase. A result of this process is that the production gas-oil ratio (GOR) rapidly increases after the critical gas saturation has been exceeded^1,2^.

Field observations in some heavy-oil reservoirs in Venezuela, Canada, and China, however, are not consistent with the solution gas drive description because the production GOR remains relatively low^3,4^. In addition, the oil production rates and oil recovery were reported to be significantly higher than the oil production rates and oil recovery that are expected from solution gas drive theory^5–7^. The most plausible explanation of the unusual production behavior is “foamy-oil” flow^8–12^, which is a different form of two-phase flow, in which the released gas flows in the form of dispersed bubbles instead of flowing as a continuous phase.

Since the conventional oil resources are dwindling, efficient exploitation of this type of heavy oil reservoir is imperative. Numerous efforts have been made to study foamy solution-gas drive processes^13–22^. Understanding the foamy oil mechanism and accurately modeling the foamy solution-gas drive processes in heavy oil reservoirs would improve the planning of the recovery of heavy oil.

In their experiments, Kumar *et al*.^13^ concluded that high oil recovery can be achieved with high depletion rates. Zhang *et al*.^14^ experimentally investigated foamy solution-gas drive processes with various temperatures and discovered that an intermediate temperature is beneficial for generating high oil recovery. Tang *et al*.^15^ investigated the effect of overburden pressure by comparing foamy solution-gas drive in consolidated rock and foamy solution-gas drive in unconsolidated rock. They discovered that overburden pressure influences the features of flowing gas bubbles, critical gas saturation and oil recovery. Alshmakhy and Maini^16^ reported an experimental study of the effect of gravity on oil recovery. They determined that gravity has a beneficial effect on recovery in some conditions because it increases the mobilizing force that is exerted on gas bubbles and encourages foamy oil flow.

In addition to these factors, oil, gas and rock properties also influence foamy solution-gas drive. High permeabilities and long sandpacks cause high supersaturation, high critical gas saturation, and high oil recovery^17^. An increase in initial water saturation causes a decreases in gas-bubble density, which produces low oil mobility and high gas mobility^18^. The oil viscosity and asphaltenes help in maintaining foamy oil flow by delaying bubble coalescence. However, they have a minor influences on oil recoveries with high depletion rates^19,20^. In addition, foamy solution-gas drive processes depend on the saturated gas types. The oil recoveries are achieved with CH_4_ saturated oil are higher than the oil recoveries achieved with CO_2_ saturated oil^21,22^.

Numerical simulation offers another effective pathway for the study of foamy solution-gas drive processes. In the early stages of research, conventional solution-gas drive models with empirical adjustments were employed to simulate foamy oil phenomena^23^. These models can be used to obtain a history match of reservoir performance. However, unrealistic parameters are often obtained due to unreasonable adjustments that account for the contributions of foamy-oil flow to oil recovery. Therefore, the predictions from these models are questionable.

Then, three types of equilibrium models were proposed based on the assumption of complete equilibrium between the gas phase and the oil phases: pseudo bubble point models^24–26^, modified fractional-flow models^27–29^ and reduced viscosity models^30^. Foamy oil is a non-thermodynamically stable system^31^. If time is sufficient and an environment is helpful, the dispersed gas will separate from the oil phase^32,33^. Thus, the equilibrium models are inherently incapable of reflecting this thermodynamically unstable characteristic of foamy oil and cannot be employed to simulate foamy oil flow.

Some kinetic models have been employed in foamy oil simulation studies to capture the time-dependent characteristic of a foamy oil system. For example, Coombe and Maini^34^ proposed a kinetic model that accounts for the changes of foamy oil morphology. Sheng *et al*.^29^ described a foamy oil model that includes two kinetic equations that control the transfers among three gas components: solution gas, evolved gas and free gas. Shahvali and Pooladi-Darvish^35^ proposed a one-dimensional dynamic model that considers the effects of viscous forces on gas mobility. In recent studies, Liu *et al*.^36^ proposed a new mathematical model for foamy-oil flow that consider foamy-oil supersaturation. Oskouei *et al*.^37^ developed a kinetic model that applies a non-equilibrium mass transfer approach to model gas evolution in heavy oil.

These models are capable of simulating the one-dimensional kinetic behavior of foamy oil flow by the history matching production behavior of pressure depletion tests. However, they are unable to predict foamy solution-gas drive processes on an oilfield scale. In addition, the models are unable to simultaneously simulate multiple characteristics of foamy solution-gas drive. The effect of microscopic behavior, such as gas bubble nucleation and growth, on foamy solution-gas drive processes is not well understood. Therefore, a new three-dimensional (3D) foamy oil model that captures many important characteristics of foamy solution-gas drive (non-equilibrium behavior, gas evolution kinetics, and the effect of viscous forces on gas mobility) based on pressure depletion tests was developed in this study. Then, the effects of some important parameters on foamy solution-gas drive were systematically investigated, and a model application was conducted in a typical foamy oil reservoir.

## Experimental and Modeling

### Experimental materials

A crude oil from the Orinoco heavy oil belt, which is located east of Venezuela was employed in the pressure depletion tests. The live oil was obtained by mixing crude oil and gas (11.1% carbon dioxide +88.9% methane). The methane gas and carbon dioxide both have purities of +99%. The physical properties of Venezuelan oil are listed in Table 1. As previously stated, the heavy oil in this area has foamy oil tendencies.

**Table 1:** Summary of the Venezuelan oil used in the experiments.

Fluid property	
GOR (m^3^/m^3^)	15
Oil density at 20 °C (g/cm^3^)	1.013
Oil viscosity at 50 °C (Pa•s)	24.7
Oil FVF at 54.2 °C and 8.65 MPa (m^3^/m^3^)	1.173
Density at 54.2 °C and 8.65 MPa (g/cm^3^)	0.957
Acid number (mg/g)	4.95
Bubble point pressure (MPa)	4.95 MPa
Pseudo bubble point pressures (MPa)	3.44 when the resting time is 24 hours
	2.74 when the resting time is 12 hours
	1.89 when the resting time is 1 hour
Monophasic fluid (mol%)	
Nitrogen	0.13
Carbon Dioxide	2.8
Methane	22.43
Propane	0.08
I-Butane	0.04
N- Butane	0.04
I-Pentane	0.12
N- Pentane	0.49
C_7+_	73.87
MW	418.76
SARA analysis (wt.%)	
Asphaltenes	7.78
Saturates	17.19
Aromatics	39.17
Resins	35.86

### Experimental setup

**Figure 1:**
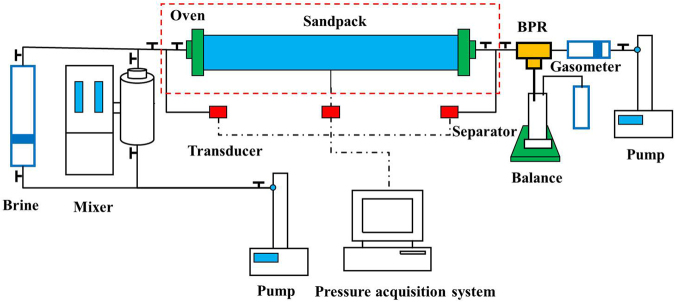
Schematic of the pressure depletion experimental setup.

A schematic of the pressure depletion experimental setup is illustrated in Fig. 1. The length and diameter of the sandpack was 50 cm and 3.8 cm, respectively; the sandpack was horizontally located in a thermostatic convection oven. The accuracy of the temperature control was ±0.1 °C. Three pressure taps monitored the pressure distribution of the sandpack. A computer was used to record the pressures from the transducers during each test.

Brine (0.5% NaCl) was contained in a transfer vessel, and live oil was obtained by saturating the crude oil with the gas in a mixer connected to the inlet end of the sandpack. The brine and live oil were injected into the sandpack by a displacement pump to saturate it prior to each test.

A displacement pump that connects a backpressure regulator (BPR) through a buffer piston vessel with water was used to control the pressure at the outlet end of the sandpack. The fluid was produced and flowed into an oil-gas separator that was located on an electronic balance to measure the produced oil. The separated gas was monitored by a gas meter that was connected to the oil-gas separator.

### Experimental procedures

According to the solution GOR (15 m^3^/m^3^) of the heavy oil, live oil at reservoir conditions (54.2 °C and 8.65 MPa) was prepared with crude oil and gas. The gas Z factor for the preparation of live oil was 0.915; it was measured by several separator tests.

After packing, the sandpack was evacuated by a vacuum pump. Then, the porosity, permeability and oil saturation of the sandpack were obtained by filling it with brine and live oil. The pressure depletion test was started after the sandpack was saturated with the live oil. The pressure was continuously decreased by discharging water from the buffer piston vessel that was connected to the displacement pump. The water discharged at a constant volume flow rate which provided a fixed depletion rate. Due to the decrease in the sandpack pressure, the fluids produced from the sandpack flowed into the oil-gas separator, and the weight of oil and the volume of gas were periodically determined by the electronic balance and the gas meter. The test was finished when the sandpack pressure declined to a relatively low value (near atmospheric pressure).

During this experimental process, the pressures at different locations were monitored with the pressure transducers. The oven temperature was maintained at a reservoir temperature of 54.2 °C. To investigate the effect of depletion rate on foamy solution-gas drive processes, three series of tests with various depletion rates were performed in our study. The properties of the sandpacks and experimental conditions are listed in Table 2.

**Table 2:** Properties of the sandpacks and the experimental results at different depletion rates.

Test No.	Porosity (%)	Oil saturation (%)	Permeability (D)	Depletion rate (MPa/h)	Cumulative oil produced (g)	Maximum oil production rate (g)	Maximum cumulative GOR (m^3^/m^3^)	Oil recovery (%)
1	42	96	7.16	0.4	37.4	5.07	90.5	17.2
2	42	96	7.11	0.8	44.3	13.07	70.2	19.1
3	41	98	7.06	1.6	63	17.87	45.6	24.6

## Modeling

### Model assumptions

The major assumptions in deriving the 3D foamy oil model are as follows:

1. The foamy oil system is considered to have two phases (oil and gas) and three components (solution gas, free gas, and oil). After gas bubble nucleation, liberation of solution gas in the oil phase is transferred to free gas in the gas phase. This process is described in the continuity equations by the sink/source term *q*_sf_, which is calculated using an equation that considers the kinetics of gas bubble nucleation and growth.
2. The density of the solution gas is equivalent to the density of the free gas, and the velocity in the solution gas in oil phase is equivalent to the velocity in the velocity in the oil phase.
3. The oil and gas relative permeabilities obey Corey-type functions, which incorporate the effects of the velocity and the oil viscosity on gas mobility.
4. The gravity effect is considered in the model, whereas the capillary effect is neglected.
5. The reservoir rock is slightly compressible.
6. The reservoir temperature is isothermal.

### Mathematical model

According to the mass balance for each component, the continuity equations are derived as follows:

1 $${\rm{Oil}}\,{\rm{component}}:\nabla \cdot [\frac{k{k}_{ro}}{{\mu }_{o}{B}_{o}}\nabla (p-{\rho }_{o}\,gH)]+{q}_{vo}=\frac{\partial }{\partial t}(\frac{\varphi {S}_{o}}{{B}_{o}})$$

2 $${\rm{Solution}}\,{\rm{gas}}\,{\rm{component}}:\nabla \cdot [{R}_{s}\frac{k{k}_{ro}}{{\mu }_{o}{B}_{o}}\nabla (p-{\rho }_{o}\,gH)]-{q}_{sf}+{R}_{s}{q}_{vo}=\frac{\partial }{\partial t}(\frac{\varphi {R}_{s}{S}_{o}}{{B}_{o}})$$

3 $${\rm{Free}}\,{\rm{gas}}\,{\rm{component}}:\nabla \cdot [\frac{k{k}_{rg}}{{\mu }_{g}{B}_{g}}\nabla (p-{\rho }_{g}gH)]+{q}_{sf}+{q}_{vg}=\frac{\partial }{\partial t}(\frac{\varphi {S}_{g}}{{B}_{g}})$$

where *k* is the absolute permeability, D; *k*_rg_ and *k*_ro_ are the gas relative permeability and oil relative permeability, respectively; *p* is the reservoir pressure, MPa; *ρ*_g_ and *ρ*_o_ are the gas density and oil density, respectively, kg/m^3^; g is the acceleration of gravity, m/s^2^; *H* is the standard height, m; *μ*_g_ and *μ*_o_ are the gas viscosity and oil viscosity, mPa·s; *S*_g_ and *S*_o_ are the gas saturation and oil saturation, respectively; *ϕ* is the porosity; *B*_g_ and *B*_o_ are the gas formation volume factor and the oil formation volume factor, respectively, m^3^/m^3^; *R*_s_ is the solution GOR, m^3^/m^3^; *q*_vo_ and *q*_vg_ are the oil volumetric rate and the gas volumetric rate, respectively, per unit bulk volume, m^3^/(m^3^·s); *t* is time, s; and *q*_sf_ is the rate of gas liberation from solution, cm^3^/s.

The oil and gas saturations satisfy the following equation:

4 $${S}_{g}+{S}_{o}=1$$

The necessary initial and boundary conditions are specified as follows:

5 $${\rm{The}}\,{\rm{initial}}\,{\rm{conditions}}:\,p(t){|}_{t=0}={p}^{0}{S}_{o}(t){|}_{t=0}={S}_{o}^{0}{S}_{g}(t){|}_{t=0}={S}_{g}^{0}$$

6 $${\rm{The}}\,{\rm{outer}}\,{\rm{boundary}}\,{\rm{condition}}:\,\frac{\partial p}{\partial n}{|}_{{\rm{\Gamma }}}=0$$

7 $${\rm{The}}\,{\rm{inner}}\,{\rm{boundary}}\,{\rm{condition}}:\begin{array}{rcl}p({x}_{w},{y}_{w},{z}_{w},t) & = & {p}_{wf}({x}_{w},{y}_{w},{z}_{w})\\ {\rm{or}}\,\,\,Q({x}_{w},{y}_{w},{z}_{w},t) & = & Q({x}_{w},{y}_{w},{z}_{w})\end{array}$$

where *P*^0^ is the initial reservoir pressure, MPa; *S*_o_^0^ and *S*_g_^0^ are the initial oil saturation and gas saturation, respectively; Γ represents the outer boundary; *n* represents the normal direction of the boundary; *p*_wf_ is the bottom-hole pressure of the production wells, MPa; *Q* is the production rate of the production wells, m^3^/d; and *x*_w_, *y*_w_, and *z*_w_ are the 3D coordinates of the production wells in the *x* direction, *y* direction and *z* direction, respectively. Eq. (1) – Eq. (7) complete the mathematical description of foamy oil model.

### Calculation of *q*_sf_

Gas bubble nucleation is assumed to occur when the supersaturation Δ*p* exceeds the capillary forces of a crevice^38^. Therefore, a crevice of radius *r*_p_ is activated when the inequality defined in the following equation is satisfied:

8 $${\rm{\Delta }}p={p}_{g}-{p}_{o}={p}_{e}-p\ge \frac{2\gamma }{{r}_{p}}$$

where *γ* is the interfacial tension, dyne/cm; *p* is the actual pressure within an oil-filled pore, MPa; *p*_e_ is the equilibrium pressure, MPa; and *p*_g_ and *p*_o_ are pressures in the gas and the oil, respectively, MPa. In this model, *r*_p_ is assumed to be randomly distributed in the porous media, which is quantitatively described using the Rayleigh distribution function *F*(*r*_p_):

9 $$F({r}_{p})=\frac{{r}_{p}}{{\sigma }^{2}}\exp (-\frac{{{r}_{p}}^{2}}{2{\sigma }^{2}})$$

In this function, the Rayleigh distribution deviation *σ* is an important parameter to determine the distribution of *r*_p_. Assume that the value of supersaturation at a specific time is represented by Δ*p*_1_, at this level of supersaturation, the pores that contain activated sites are pores with a radius larger than *r*_pmin_ (*r*_pmin_ is equal to 2γ/Δ*p*_1_ based on Eq. (8)). Gas bubble nucleation occurs in pores with radii between *r*_pmin_ and the maximum radius *r*_pmax_. Therefore, the number of bubbles per unit bulk volume *N* can be obtained by the following equation:

10 $$N=\frac{\varphi {\int }_{{r}_{pmin}}^{{r}_{pmax}}F({r}_{p})d{r}_{p}}{{V}_{s}}$$

where *V*_s_ is the average volume of one pore (assuming the existence of one nucleus bubble in each pore). The growth of the activated gas bubbles is controlled by diffusion of the solution gas in the supersaturated oil. Therefore, the rate of gas bubble growth is^39^:

11 $$\frac{dr}{dt}=\frac{DRT{\rm{\Delta }}C}{pr}$$

where *r* is the gas bubble radius, m; *D* is the diffusion coefficient, m^2^/s; R is the universal gas constant, J/(mol·K); Δ*C* is the supersaturation expressed as a difference in concentration, mol/m^3^; and *T* is the temperature, K.

If the radius of a gas bubble is increased from *t*^n^ to *t*^n+1^, *r* at time *t*^n+1^ is:

12 $$r({t}^{n+1})=r({t}^{n})+\frac{dr}{dt}{\rm{\Delta }}t$$

where Δ*t* is the increment of time from *t*^n^ to *t*^n+1^, s.

If the gas bubbles are assumed to be spherical, the total volume of the gas bubbles per unit bulk volume is:

13 $$V=N\frac{4}{3}\pi {r}^{2}$$

The volume increase of the gas bubbles from *t*^n^ to *t*^n+1^ is:

14 $$\frac{dV}{dt}=\frac{N4\pi {r}^{2}dr}{dt}=\frac{N4\pi DRT{\rm{\Delta }}C(t)r({t}^{n})}{p}$$

For a pre-existing bubbles nucleation model, where new bubbles at different population densities continuously activate, Eq. (14) should be changed to the following form:

15 $$\frac{dV}{dt}=\frac{N4\pi {r}^{2}dr}{dt}=\frac{4\pi DRT{\rm{\Delta }}C(t)}{p}\sum _{j}{N}_{j}{r}_{j}$$

where the subscript *j* denotes the *j*^*th*^ time step in which new nuclei at a density of *N*_*j*_ are activated; and *r*_*j*_ is the radius of a gas bubble at the *j*^*th*^ time step.

The volumetric rate of gas liberation from solution per unit bulk volume at standard conditions can be calculated by:

16 $${q}_{sf}=\frac{dV}{dt}\frac{1}{{B}_{g}}=\frac{4\pi DRT{\rm{\Delta }}C(t)}{p{B}_{g}}\sum _{j}{N}_{j}{r}_{j}$$

The gas concentration can be related to the solution GOR using the following equations:

17 $${\rm{\Delta }}C(t)=C-{C}_{eq}=\frac{{R}_{s}{\rho }_{gsc}}{{B}_{o}}-\frac{{R}_{seq}{\rho }_{gsc}}{{B}_{o}}$$

where *C* is the average gas concentration in the liquid phase, mol/m^3^; *C*_*eq*_ is the equilibrium gas concentration, mol/m^3^; *R*_seq_ is the equilibrium solution GOR, m^3^/m^3^; and *ρ*_gsc_ is the gas molar density at standard conditions.

Eq. (18) is the final equation applied in the model to account for gas evolution from solution:

18 $${q}_{sf}=\frac{4\pi DRT(R-{R}_{eq}){\rho }_{gsc}}{p{B}_{o}{B}_{g}}\sum _{j}{N}_{j}{r}_{j}$$

### Oil-gas relative permeabilities

During foamy solution-gas drive processes, gas is dispersed in the oil phase to generate foamy oil due to the high viscous forces of heavy oil, which cause a decrease in the gas mobility^40–42^. Thus, oil and gas relative permeabilities depend not only on gas saturation but also on viscous forces. In this study, the effect of viscous forces on gas mobility are represented by oil and gas relative permeabilities, which are given by the following equations^38^:

19 $${k}_{ro}={k}_{ro}^{0}{(\frac{1-{S}_{g}}{1-{S}_{gc}})}^{{n}_{o}}\,{S}_{gc}={S}_{gci}+\beta v{{\mu }_{o}}^{1/2}$$

20 $${k}_{rg}={k}_{rg}^{0}{(\frac{{S}_{g}-{S}_{gc}}{1-{S}_{gc}})}^{{n}_{g}}\,{k}_{rg}^{0}={k}_{rgi}\exp (-\alpha v{{\mu }_{o}}^{\frac{1}{2}})$$

where *S*_*gc*_ is the critical gas saturation; *n*_*o*_ is the Corey exponent for oil; *n*_*g*_ is the Corey exponent for gas; *k*_*rg*_^0^ and *k*_*ro*_^0^ are the end-point gas and oil relative permeability, respectively; *k*_*rgi*_ is the maximum value of gas relative permeability; *S*_*gci*_ is the minimum value of critical gas saturation; *α* and *β* are proportionality constants, s/m; and *v* is the oil phase velocity, m/s.

### Other properties

Oil and gas production rates:

21 $${q}_{vo}=\frac{Q}{{V}_{B}{B}_{o}(1+(\frac{{k}_{rg}}{{k}_{ro}})(\frac{{\mu }_{o}}{{\mu }_{g}}))}\,{q}_{vg}=\frac{Q(\frac{{k}_{rg}}{{k}_{ro}})(\frac{{\mu }_{o}}{{\mu }_{g}})}{{V}_{B}{B}_{g}(1+(\frac{{k}_{rg}}{{k}_{ro}})(\frac{{\mu }_{o}}{{\mu }_{g}}))}$$

22 $${\rm{Gas}}\,{\rm{FVF}}:\,{B}_{g}=\frac{T{p}_{sc}Z}{p{T}_{sc}}$$

23 $${\rm{Oil}}\,{\rm{FVF}}:{B}_{o}={B}_{ob}[1+{C}_{o}(p-{p}_{b})]\,\,p\ge {p}_{b}$$

24 $${B}_{o}=1+\frac{{B}_{ob}-1}{{p}_{b}}p\,p < {p}_{b}$$

where *V*_B_ is the volume of a grid block, cm^3^; *Z* is the compressibility factor; *p*_sc_ is the standard pressure, MPa; *p*_*b*_ is the bubble point pressure, MPa; *B*_*ob*_ is the oil formation volume factors at bubble point pressure; *C*_*o*_ is the oil compressibility coefficient, 1/MPa; and *T*_sc_ is the standard temperature, °C.

### Solution of the mathematical model

The model Eqs (1–7) were solved using the IMPES method. First, the continuity equations for the three components multiplied by appropriate coefficients were merged into a pressure equation. In a 3D model with *Nx*, *Ny*, and *Nz* blocks, 3*N* pressure equations were generated. The system was solved by a conjugate gradient method for the pressure of each block. Once the pressures were obtained, *S*_g_ and *R*_s_ were explicitly obtained, and *S*_o_ was calculated by Eq. (4). To efficiently and accurately solve the mathematical model, we developed a 3D two-phase flow numerical simulator.

## Results and Discussion

### Experimental results

**Figure 2:**
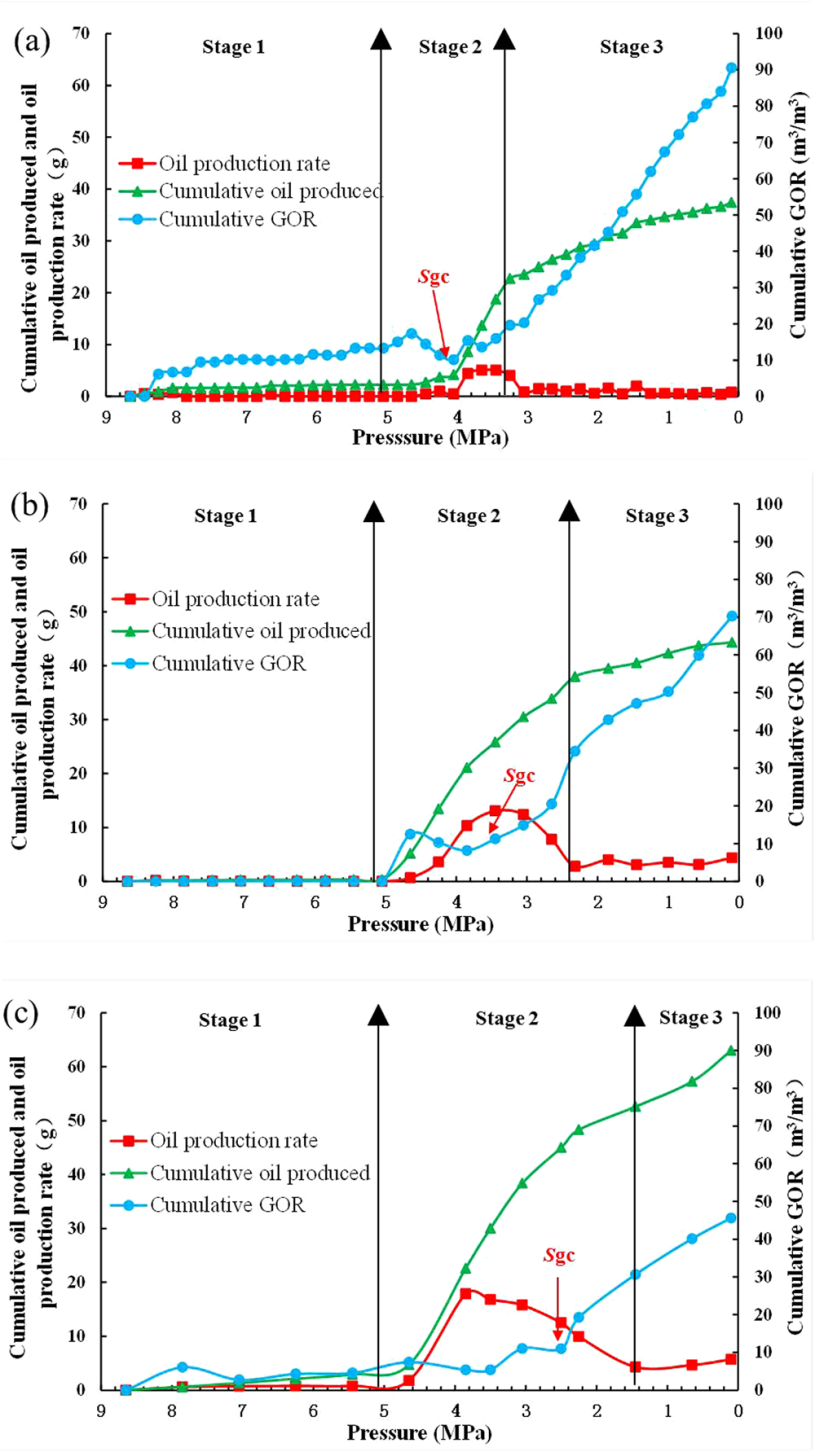
Cumulative oil produced, cumulative GOR and oil production rate vs. pressure for the three pressure depletion tests: (**a**) 0.4 MPa/h; (**b**) 0.8 MPa/h; (**c**) 1.6 MPa/h.

Figure 2 and Table 2 provide the experimental results for different depletion rates. The pressure in Fig. 2 is the average pressure of the sandpack. Figure 2 shows three typical stages for the foamy solution-gas drive in all tests. In stage 1, when the pressure exceed *P*_b_ (4.95 MPa), the cumulative oil produced, the cumulative GOR and the oil production rate are very low. Because the solution gas is not released from the oil and the fluid in the sandpack flowed as a single oil phase in this stage. The oil is primarily produced by the expansion of the low compressibility oil phase. However, once the pressure is below *P*_b_ (Stage 2), the cumulative GOR remains nearly constant for a period of time, and the cumulative oil produced and oil production rate rapidly increase. The unusual performances are caused by the effect of foamy oil flow. When the sandpack pressure was below *P*_b_, the gas was released from solution and dispersed in the oil as tiny bubbles rather than coalescing to form a continuous gas phase that causes fast gas breakthrough, energy loss and decreased oil production. The oil with dispersed bubbles (foamy oil phenomena) causes oil volume swelling to provide energy and prevent a decrease in pressure and oil production. Since the gas does not become continuous in this stage, its mobility remains low, which causes stable cumulative GOR.

As pressure depletion continues, at a certain pressure, the critical gas saturation (*S*_gc_) is attained, and the cumulative GOR starts to linearly increase with pressure, which indicates that the gas bubbles in oil begin to coalesce and form a continuous gas phase and foamy oil phenomena begin to disappear. Note that the production of foamy oil continues although free gas starts to flow at this pressure. This finding is consistent with the observations reported by Alshmakhy and Maini^16^.

In stage 3, the cumulative oil produced and oil production rate become low, and the cumulative GOR continually increases due to the complete disappearance of foamy oil phenomena in this stage. The gas bubbles trapped in the oil phase completely coalesce and generate free gas. Because the gas mobility is higher than the oil mobility, which causes gas breakthrough, a significant decrease in the cumulative oil produced and oil production rate, and an increase in the cumulative GOR. The three typical stages can be used to determine whether a heavy oil exhibits significant foamy oil tendencies during solution-gas drive processes.

As shown in Table 2, a higher depletion rate produces a higher cumulative oil produced, higher maximum oil production rate, higher oil recovery and lower maximum cumulative GOR. High depletion rates benefit foamy solution-gas drive processes. The mechanism responsible for this pronounced effect of depletion rate is ambiguous and can be related to the depletion rate dependence of *S*_gc_. The highest *S*_gc_ is achieved at the highest depletion rate (Fig. 2), which decreases the free gas mobility. In addition, high pressure depletion rates generate a large amount of supersaturation. Additional nucleation sites are activated and smaller gas bubbles are formed.

### Modeling

#### Verification of the new developed model

**Figure 3:**
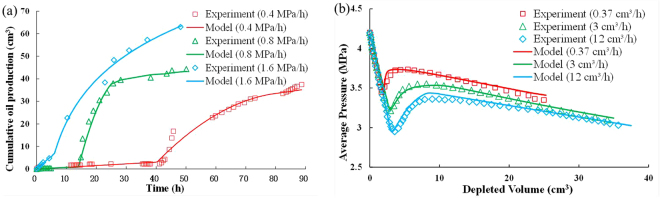
Comparison of the experimental data and simulated results: (**a**) this study; (**b**) Kumar’s study.

The proposed foamy oil model was validated by comparing the experimental data of the pressure depletion tests in this study with the experimental data of the pressure depletion tests in Kumar’s study^14^. The rock and fluid properties corresponding to the experimental data are listed in Tables 2 and 3. During the history-matching processes, the adjusted parameters in the model were parameters related to the oil and gas relative permeabilities (*S*_*gci*_, *k*_*rgi*_, *α*, and *β*), gas bubble nucleation and growth (*D*, σ, and *r*_pmax_). The results from the developed foamy model and the experiments are compared in Fig. 3. As depicted by Fig. 3, fairly reasonable matches are obtained.

**Table 3:** Values in the models before and after matching for this study and Kumar’s study.

Parameters	This study		Kumar’s study	
	Before matching	After matching	Before matching	After matching
Maximum *k*_*ro*_^0^	1	1	1	1
Maximum *k*_*rgi*_	0.1	0.06	0.08	0.05
*S*_*gci*_	0.02	0.025	0.025	0.02
*n*_*o*_	2	2	2	2
*n*_*g*_	2	2	2	2
*r*_p*max*_ (m)	1.0 × 10^−7^	1.2 × 10^−7^	0.8 × 10^−7^	1.0 × 10^−7^
*σ* (m)	2.0 × 10^−8^	1.7 × 10^−8^	1.0 × 10^−8^	1.5 × 10^−8^
*α* (s/m)	1.0 × 10^6^	1.2 × 10^6^	1.0 × 10^5^	8.4 × 10^6^
*β* (s/m)	1.0 × 10^4^	1.5 × 10^4^	1.0 × 10^4^	1.2 × 10^4^
*D* (m^2^/s)	1 × 10^−9^	3 × 10^−9^	1 × 10^−9^	3 × 10^−9^

In addition, when pressure exceeds *P*_b_, no gas bubbles were released from the oil phase to form foamy oil. At this stage, only single oil flow exists in the reservoir. Thus, the accuracy of the foamy oil model for the flow process above *P*_b_ can be also validated against the known analytical solution of a one-dimensional single-phase flow model^6^ and Eclipse’s black oil simulator E100.

**Figure 4:**
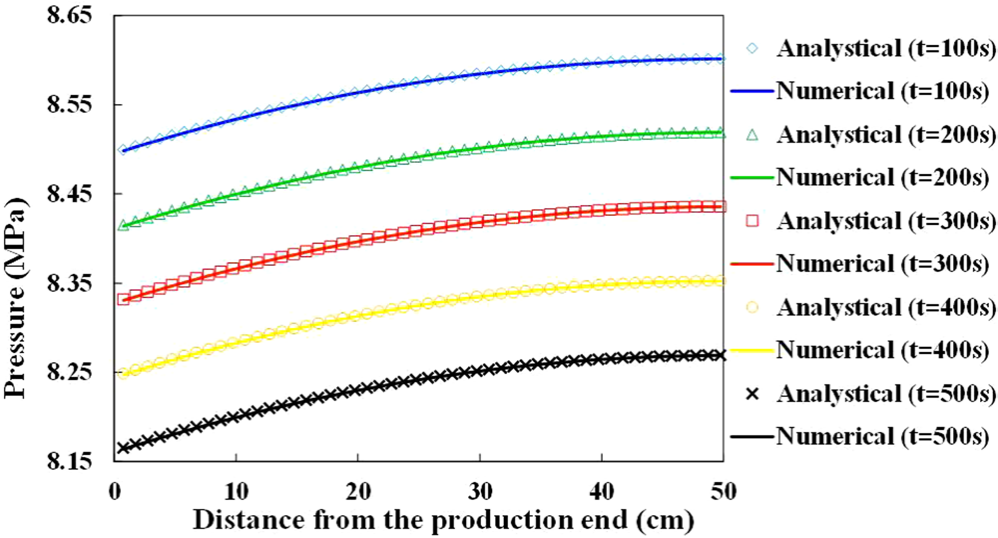
Comparison of the pressures above Pb vs. distance at different times.

**Figure 5:**
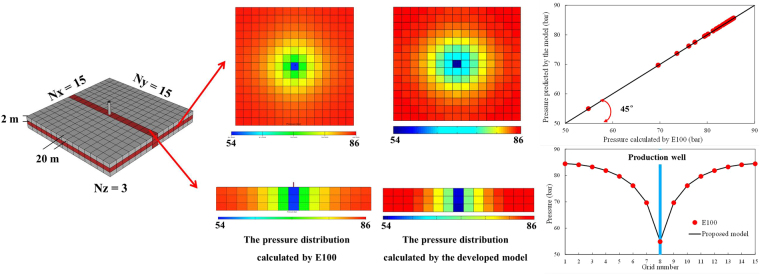
Comparison of the pressure distributions and specific pressure values.

The simulation results of pressures above *P*_b_ at different times are compared with the simulation results obtained by the analytical solution, which is presented in Fig. 4. The analytical solution matches the calculated results of the model. Comparisons of the solutions of the 3D foamy oil model and solutions obtained from E100 indicate that the average relative error in the pressure values in each grid between the 3D developed model and the E100 model is 0.103% after the simulation calculations (t = 30 days). In addition, Fig. 5 implies agreement between the calculated pressure values in a 3D reservoir obtained by the developed model and E100. The simulated results of the developed model are consistent with the results of the analytical solution and E100.

#### Study of affecting factors

The model after the experimental history-match was employed as the base case; its parameters are listed in Tables 2 and 3. To align the range of the factors analyzed with the actual conditions of the Venezuelan reservoir, the model parameters were adjusted with a constant factor of 25% based on the base case (Table 4).

1. Effect of critical gas saturation (*S*_gc_)
  As shown in Fig. 6, the higher is the oil recovery, the lower is the cumulative gas produced and the longer is the predicted time for gas produced for a higher *S*_gc_. However, the pressure is not significantly affected by *S*_gc_. Note that the definition of *S*_gc_ is the gas saturation when the gas starts to flow as a continuous phase. Thus, a high *S*_gc_ causes delay in the time for gas produced, which produces high oil recovery and low cumulative gas produced.

**Figure 6:**
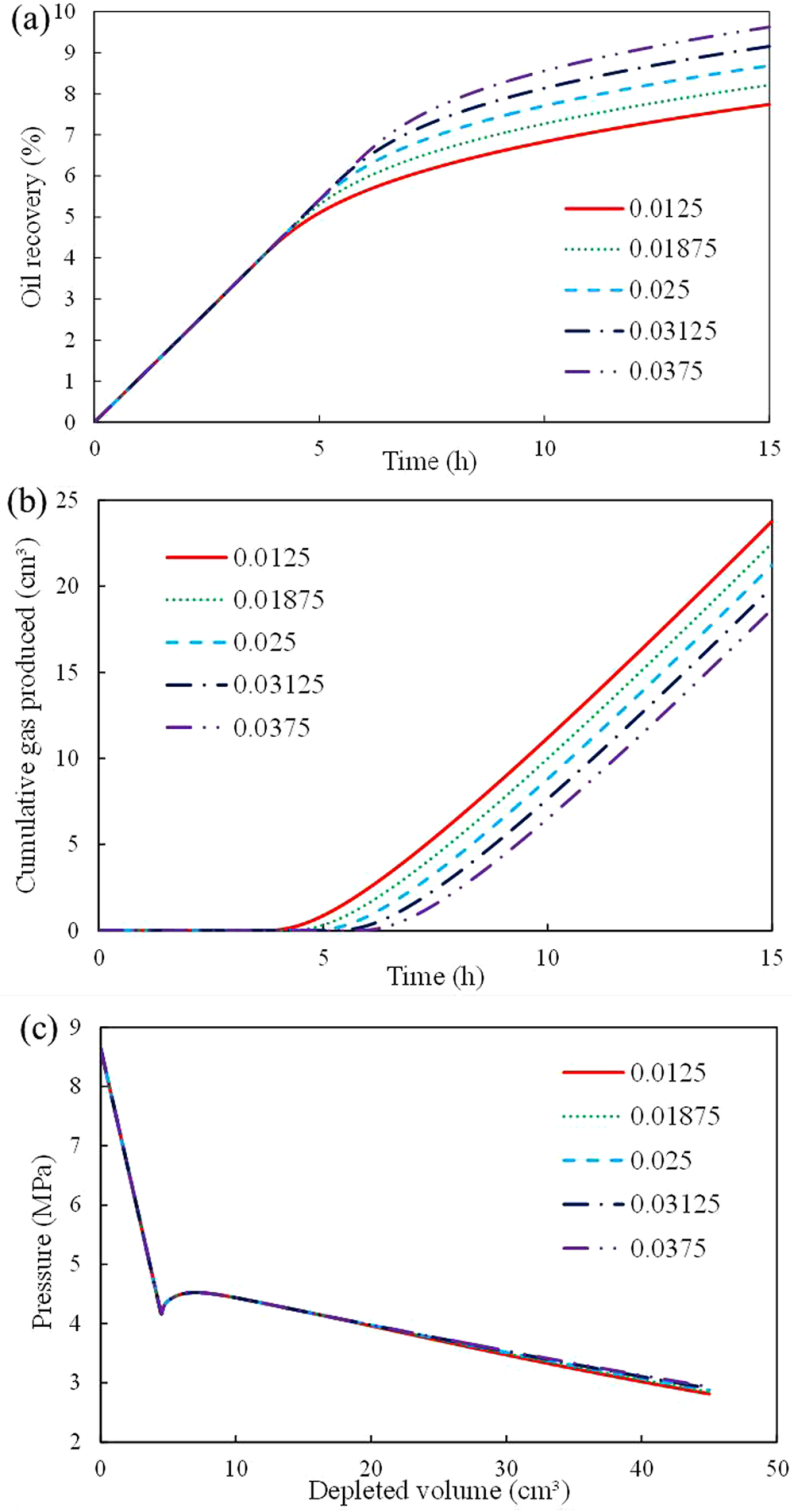
Effect of Sgc on foamy solution-gas drive processes: (**a**) oil recovery; (**b**) cumulative gas produced; (**c**)average pressure.
  In addition, the calculated *N* (the number of bubbles per unit bulk volume) and *r* (the radius of gas bubbles) in every time step from each run have the same values (the maximum *N* and minimum *r* in each run are 2.04 × 10^10^ and 8.63 × 10^−8^ m, respectively). Thus, *S*_gc_ never affects the gas bubble nucleation and growth behavior (the gas bubble nucleation and growth behavior is primarily related to the pressure), which explains why *S*_gc_ does not significantly influence the pressure profiles (Fig. 6c).
2. Effect of oil viscosity (*μ*_o_)
  As revealed by Fig. 7, increasing *μ*_o_ causes delay of the time in gas produced, a decrease in cumulative gas produced, and an increase in the oil recovery. The results are consistent with the results of an experimental study by Pooladi-darvish and Firoozabadi^19^. As shown in Fig. 7c, increasing the oil viscosity affects the early time stage of the pressure profiles. The pressure rebound is small when the oil viscosity is high because high oil viscosity inhibits the gas diffusion process, which decreases the values of *q*_sf_ (the volumetric rate of gas liberation from solution). For example, when the oil viscosities are 2086.5 mPa·s and 6259.5 mPa·s, the calculated maximum *q*_sf_ are 4.81 × 10^−4^ m^3^/(m^3^⋅s) and 2.68 × 10^−4^ m^3^/(m^3^⋅s), respectively. Thus, increasing the oil viscosity affects the early time stage of foamy solution-gas drive processes. As shown in Fig. 7b, high oil viscosity decreases the gas mobility. Increasing the oil viscosity from 2086.5 mPa·s to 6259.5 mPa·s increases the critical gas saturation *S*_gc_ from 4.09% to 5.26%, respectively. Therefore, increasing oil viscosity can also affect the late-time stage of foamy solution-gas drive processes due to the decrease in gas mobility.

**Figure 7:**
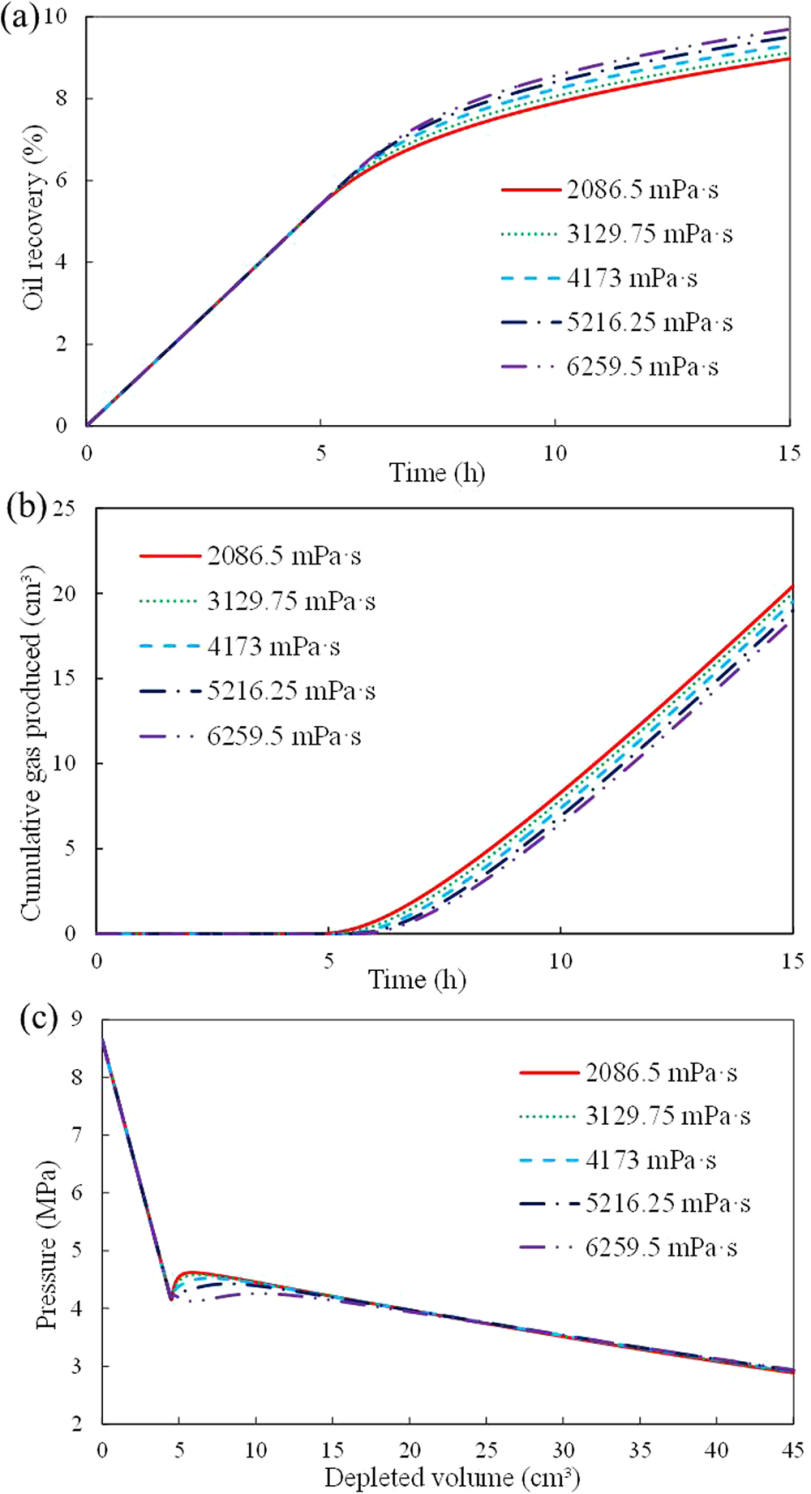
Effect of μo on foamy solution-gas drive processes: (**a**) oil recovery; (**b**) cumulative gas produced; (**c**)average pressure.
  These studies explain how oil viscosity influences foamy solution-gas drive processes. However, their findings do not explain whether a reduction in *q*_sf_ or a reduction in gas mobility enables high viscosity oil to enhance the performance of foamy solution-gas drive processes. Because the calculated value of *q*_*sf*_ continuously changes during the simulation processes, the effect of *q*_*sf*_ on foamy solution-gas drive processes can not be directly investigated by comparing the results of different runs with fixed *q*_sf_ values. According to Eq. (18), the diffusion coefficient *D* is positively proportional to *q*_sf_, and it does not affect gas mobility. Therefore, *D* can be applied to indirectly investigate the effect of *q*_sf_ on foamy solution-gas drive processes.
  The results indicate that an increase in *D* decreases the non-equilibrium effects and the supersaturation level in the early time of the average pressure profiles because a high *D* facilitates the growth of gas bubbles, and gas liberates from solution at a high rate, which is equivalent to increasing the value of *q*_sf_. However, the cumulative gas produced and oil recovery are insensitive to changes in *D* for the range of the study. Therefore, we infer that oil viscosity affects foamy solution-gas drive by decreasing gas mobility, rather than inhibiting the growth of gas bubbles (decreasing the *q*_sf_).
3. Effect of depletion rate
  The simulated results indicate that efficient oil recovery is obtained at higher depletion rates, which indicates that high depletion rates are beneficial to foamy solution-gas drive processes. These predictions are consistent with the experimental observations for the following reasons:
  First, the higher is the pressure depletion rate is, the larger is the formed supersaturation. Therefore, a larger number of nucleation sites are activated and smaller gas bubbles are formed. For example, when the pressure depletion rates are 1.5 m^3^/h and 4.5 m^3^/h, the calculated maximum *N* (the number of bubbles per unit bulk volume) are 8.13 × 10^9^ and 8.66 × 10^10^, respectively, and the calculated minimum *r* (the radius of gas bubbles) are 9.14 × 10^−8^ m and 8.01 × 10^−8^ m, respectively. A larger number of activated nucleation sites cause a greater expansion energy of heavy oil, and smaller bubbles are easily trapped in the oil phase. Therefore, foamy oil is more effective and stable. In addition, high depletion rates cause an increase in *S*_gc_. Increasing the depletion rate from 1.5 m^3^/h to 4.5 m^3^/h causes an increase in *S*_gc_ from 3.63% to 5.88%. Thus, the free gas mobility decreases, which causes a delay in the time of gas produced and increase the cumulative gas produced. We know that the pressure depletion rate not only influences the early time stage of gas bubble nucleation and growth but also affects the late-time stage of gas flow.
4. Effect of scale
  The effect of scale was investigated by simulating pressure depletion tests in sandpacks of different lengths. Figure 8a presents the calculated oil recoveries from the sandpacks of different lengths, which shows that the oil recovery increases as the length decreases. The length primarily affects the pressure gradient within the sandpacks. In the short sandpack, a high pressure gradient develops in the entire region of the sandpack. Therefore, the pressure depletion rate for the shorter sandpack is faster than the pressure depletion rate for the longer sandpack (Fig. 8c), which generates more efficient oil recovery. In addition, the higher pressure depletion rate for the shorter sandpack causes fast gas bubble nucleation, growth and gas produced. This finding explains why the cumulative gas produced in the shorter sandpack is higher than the cumulative gas produced in the longer sandpack (Fig. 8b).

**Figure 8:**
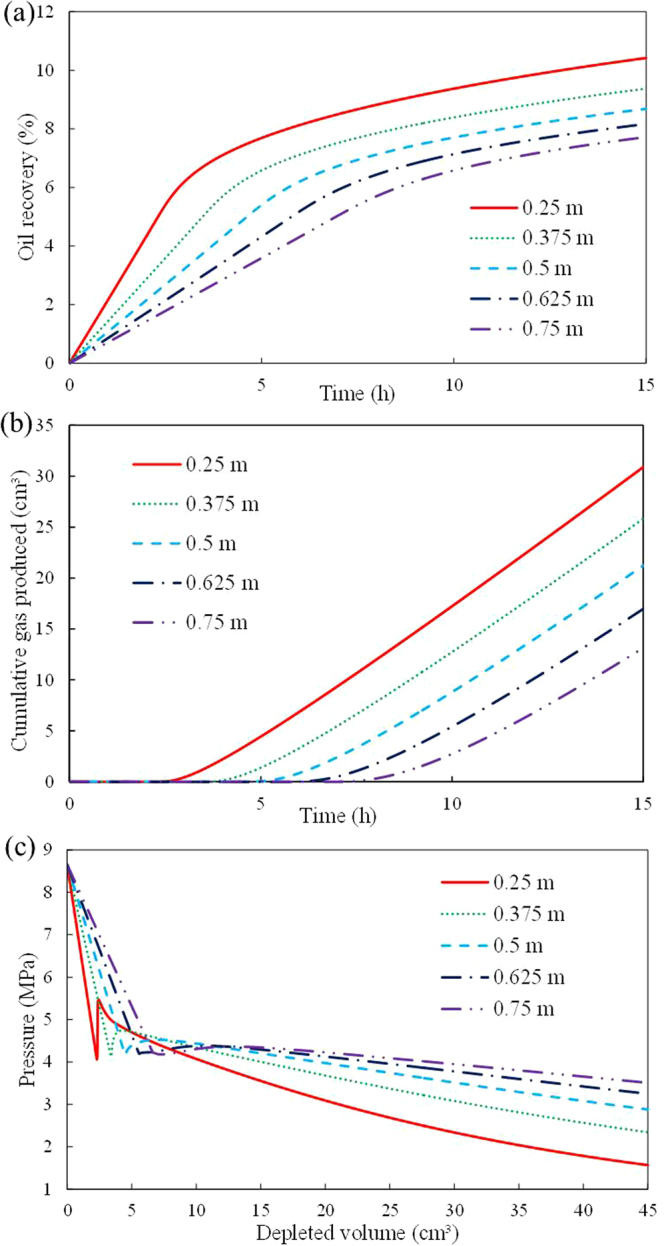
Effect of sandpack length on foamy solution-gas drive processes: (**a**) oil recovery; (**b**) cumulative gas produced; (**c**) average pressure.
5. Effect of solution GOR

**Table 4:** Affecting factors investigated in this study and their effects on foamy solution-gas drive processes.

Affecting factors	Range of affecting factors					Change of factors (times)	Change of oil recovery (%)	Change of cumulative gas produced (%)
Depletion rate (cm^3^/h)	1.5	2.25	3	3.75	4.5	3	45.79	53.28
Scale (length) (m)	0.25	0.375	0.5	0.625	0.75	3	25.89	122.33
Critical gas saturation	0.0125	0.01875	0.025	0.03125	0.0375	3	21.46	24.35
Oil viscosity (mPa•s)	2086.5	3129.75	4173	5216.25	6259.5	3	9.52	8.02
Solution GOR (m^3^/m^3^)	7.8	11.7	15.6	19.5	23.4	3	4.82	5.86
Diffusion coefficient (m^2^/s)	1 × 10^−11^	5 × 10^−11^	1 × 10^−10^	5 × 10^−10^	1 × 10^−9^	100	0.64	0.26

The simulated results of depletion tests with oil of varying solution GOR reveal that a high solution GOR causes low oil recovery, high cumulative gas produced and high pressure. The solution GOR exerts a significant influence on the sandpack pressures. However, the effect of solution GOR on oil recovery and cumulative gas produced is not distinct. When the heavy oil has a high solution GOR, the rate of gas bubble growth is high, and more gas released from solution starts to flow as a continuous phase. As a result, the gas saturation in the sandpack increases at a faster rate. When the gas saturation attains *S*_gc_, the free gas begins production. Therefore, the high solution GOR causes low oil recovery and high cumulative gas produced, which indicates that maintaining the foamy oil flow becomes more difficult when the solution GOR of heavy oil is too high. However, when the heavy oil has a high solution GOR, the high rate of gas bubble growth causes significant expansion of the heavy oil, which is beneficial for maintaining the sandpack pressure.

#### Sensitive factors

The previous discussion of the affecting factors indicates that foamy solution-gas drive processes are very complicated, and can be affected by many factors. However, a systematic and quantitative study has determine the sensitive factors for foamy solution-gas drive processes. In this study, all affecting factors were adjusted with a constant factor of 25%. Thus, the variation in the factors investigated in this study remains constant (Table 4). The maximum values are increased by three times compared with their minimum values. In this manner, a comparison of the performance of these cases can provide information about the sensitive factors for foamy solution-gas drive.

Depending on the extent of change shown in Table 4, the simulated results indicate that the corresponding change in oil recovery for the depletion rate, length, and *S*_gc_, *μ*_o_, and GOR are 45.79%, 25.89%, 21.46%, 9.52%, and 4.82% respectively, and the change in cumulative gas produced for the depletion rate, length, and *S*_gc_, *μ*_o_, and GOR are 53.28%, 122.33%, 24.35%, 8.02% and 5.86%, respectively. Therefore, the oil recovery is sensitive to the depletion rate, length, and *S*_gc_, and the cumulative gas produced is sensitive to the length, depletion rate, and *S*_gc_. Oil viscosity and solution GOR are not sensitive factors for foamy solution-gas drive processes. Although the values of the diffusion coefficient increased by 100 times, this coefficient has a minimal effect on foamy solution-gas drive processes (the change in oil recovery and the change in cumulative gas produced are 0.64% and 0.26%, respectively).

#### Model application in M reservoir

**Figure 9:**
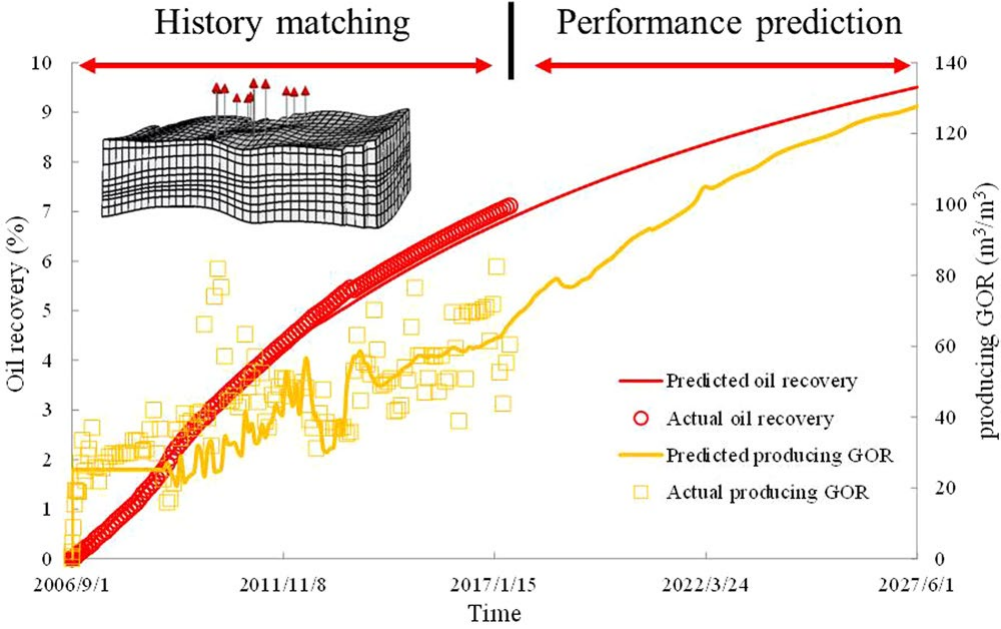
Results of history matching and performance prediction for foamy solution-gas drive of a typical production platform.

The M reservoir is located along the southern margin of the Eastern Venezuela Basin. The developed foamy oil model was used to predict the performance of a production platform in the M reservoir in foamy solution-gas drive processes (Fig. 9). The controlled region of the platform is 3200 m × 1200 m with a pay zone thickness of 200 m. The values of the porosities and permeabilities vary within the range of 3819 mD–9511 mD and 0.14–0.20, respectively. The region consists of three layers with four wells in each layer. The well spacing and row spacing are 600 m. This region is simulated with 3780 gridblocks. Timesteps are automatically controlled with a maximum allowable timestep size of one day. This limit is chosen due to accuracy considerations.

Because the pressure depletion tests conducted in this study were performed to simulate the cold production of this region, the oil sample was obtained from this region, and the foamy oil model parameters for this simulation are directly applied after an experimental match was obtained (Table 3). The only uncertain parameters in this model are geologic parameters. To ensure the accuracy of these parameters, production history matching was conducted using the developed model, which is shown in Fig. 9.

As shown in Fig. 9, the predicted oil recovery rapidly increased, and the producing GOR remained relatively stable. Therefore, the predicted results calculated by the developed model can reflect the production characteristics of foamy solution-gas drive process. Thus, the model is effective for simulating the foamy solution-gas drive process for an actual reservoir.

After the history matching process, the performance prediction of the platform was conducted by the model. The results reveal that the ten-year oil recovery of foamy solution-gas drive production was 9.51%. The increased extent of oil recovery gradually reduces, and the producing GOR sharply increased from 66.42 m^3^/m^3^ to 127.65 m^3^/m^3^ in the next ten years because gas bubbles trapped in oil phase coalesce to form free gas with a decrease of reservoir pressure, which causes the disappearance of foamy oil mechanism. The foamy oil flow will become a two phase flow of oil and gas in the reservoir in the next ten years. Therefore, enhanced oil recovery methods are needed to extend the foamy solution-gas drive process and increase the oil recovery of the platform.

## Conclusions

1. Three typical stages for foamy solution-gas drive can be employed to determine whether a heavy oil exhibits substantial foamy oil tendencies. Higher depletion rates generate in more activated nucleation sites, smaller gas bubbles, and higher *S*_gc_, which benefits foamy solution-gas drive processes.
2. High oil viscosity is beneficial in foamy solution-gas drive processes. Oil viscosity affects foamy solution-gas drive primarily by decreasing gas mobility rather than inhibiting the growth of gas bubbles.
3. A high pressure gradient develops in the short sandpack, which enable high oil recovery and simultaneously causes fast bubble nucleation, bubble growth and gas produced in the shorter sandpack.
4. The solution GOR exerts a strong influence on sandpack pressures. However, the effect of solution GOR on oil recovery and cumulative gas produced are not distinct.
5. Foamy solution-gas drive processes are sensitive to the depletion rate, length, and critical gas saturation. Oil viscosity, solution GOR and diffusion coefficient are not sensitive factors.
6. Our experimental results are based on ideal conditions. The real practice of developing oil in heavy oil reservoirs requires further analysis. In addition, the new developed model was only verified by pressure depletion tests and a typical foamy oil reservoir. Additional studies are needed to verify the validity of the model in more complex conditions.
